# PACAP–Sirtuin3 alleviates cognitive impairment through autophagy in Alzheimer’s disease

**DOI:** 10.1186/s13195-023-01334-2

**Published:** 2023-10-27

**Authors:** Qing Wang, Yue Wang, Shiping Li, Jiong Shi

**Affiliations:** 1 Department of Neurology, Beijing Tiantan Hospital, Capital Medical University, No. 119, South 4Th Ring West Road, Fengtai District, Beijing, 100070 China; 2 China National Clinical Research Center for Neurological Diseases, Beijing Tiantan Hospital, Capital Medical University, Beijing, China; 3 Advanced Innovation Center for Human Brain Protection, Capital Medical University, Beijing, China

**Keywords:** Alzheimer’s disease, Autophagy, PACAP, AKT, PI3K, mTOR, Sirt3

## Abstract

**Background:**

Autophagy is vital in the pathogenesis of neurodegeneration. Thus far, no studies have specifically investigated the relationship between pituitary adenylate cyclase-activating polypeptide (PACAP) and autophagy, particularly in the context of Alzheimer’s disease (AD). This study used in vitro and in vivo models, along with clinical samples, to explore interactions between PACAP and autophagy in AD.

**Methods:**

AD model mice were administered 6 μl of 0.1 mg/ml PACAP liquid intranasally for 4 weeks, then subjected to behavioral analyses to assess the benefits of PACAP treatment. The underlying mechanisms of PACAP-induced effects were investigated by methods including real-time quantitative polymerase chain reaction, RNA sequencing, immunofluorescence, and western blotting. Exosomes were extracted from human serum and subjected to enzyme-linked immunosorbent assays to examine autophagy pathways. The clinical and therapeutic implications of PACAP and autophagy were extensively investigated throughout the experiment.

**Results:**

Impaired autophagy was a critical step in amyloid β (Aβ) and Tau deposition; PACAP enhanced autophagy and attenuated cognitive impairment. RNA sequencing revealed three pathways that may be involved in AD progression: PI3K-AKT, mTOR, and AMPK. In vivo and in vitro studies showed that sirtuin3 knockdown diminished the ability of PACAP to restore normal autophagy function, resulting in phagocytosis dysregulation and the accumulation of pTau, Tau, and Aβ. Additionally, the autophagic biomarker MAP1LC3 demonstrated a positive association with PACAP in human serum.

**Conclusions:**

PACAP reverses AD-induced cognitive impairment through autophagy, using sirtuin3 as a key mediator. MAP1LC3 has a positive relationship with PACAP in humans. These findings provide insights regarding potential uses of intranasal PACAP and sirtuin3 agonists in AD treatment.

**Trial registration:**

NCT04320368.

**Supplementary Information:**

The online version contains supplementary material available at 10.1186/s13195-023-01334-2.

## Introduction

Alzheimer’s disease (AD), as a neurodegenerative disease, causes a progressively cognitive decline [1, 2]. Autophagy is a process phagocytosing and eventually degrading the cytoplasmic proteins or organelles, impaired in AD [3, 4]. Enhanced autophagic function can eliminate amyloid β (Aβ) and Tau deposition, thereby reversing cognitive impairment [5–10]. Autophagy is initiated by cellular membranes (plasma or organelle). A phagosome becomes a two-layer membrane structure, which packages cytoplasmic proteins or organelle components. The lysosome fuses with outer membrane and form an autolysosome that digests the packaged contents [11, 12] (Additional file 1: Supplementary Fig. S1A). At present, autophagy is a potential therapy target for AD [13], most believe that autophagy is weakened, while enhanced autophagy is helpful to clear up β-Amyloid deposition [5–9], and a few people believe that autophagy in AD is enhanced, and damages some normal neurons and aggravates disease process [14–17].

The phosphatidylinositol-3-kinase–serine/threonine kinase (PI3K-AKT), mechanistic target of rapamycin kinase (mTOR), and 5’adenosine monophosphate-activated protein kinase (AMPK) pathways are classic autophagy signaling pathways. Generally, the autophagic flux will be at a low level and increase PI3K-AKT, mTOR pathway can promote the growth, differentiation, and survival of cell [18]. Overexpression of the mTOR and PI3K-AKT pathways are early features of AD related to synaptic loss and cognitive decline [18–20]; AMPK upregulation can induce autophagy [21–23]. During autophagy activation, the beclin1-vacuolar protein sorting 34 (VPS34) complex [24–26] and ATG12-ATG5-ATG16 complex [27] interact with microtubule-associated protein 1 light chain 3-I (MAP1LC3-I or LC3-I). LC3-I binds to phosphatidylethanolamine (PE) and becomes LC3-II, which localizes to the autophagosome inner membrane and participates in autolysosome formation [28] (Additional file 1: Supplementary Fig. S1A). This theoretical basis supports further studies of pathways involved in autophagy.

Pituitary adenylate cyclase-activating polypeptide (PACAP) was a neuropeptide, originally obtained from sheep hypothalamus [29]. PACAP-38, the main form of PACAP in the brain, has neurotrophic and neuroregulatory effects. There is evidence that PACAP can stimulate non-amyloid processing, inhibit Aβ and Tau deposition, promote clearance of Tau and Aβ deposition, improve cognitive ability in AD mice; a lack of PACAP in brain affected the severity of AD pathology in APP/PS1/tau triple-transgenic mice (3xTg mice) [30–34]. PACAP can alleviate Aβ toxicity through the deacetylase sirtuin3 (Sirt3) [35, 36]. Thus far, there is limited effective treatment for AD; new treatments are urgently needed [37]. PACAP plays an antiapoptotic role and regulates synaptic plasticity through Sirt3 in hypoperfusion model [38]. In addition, PACAP has important neuroprotective roles in various diseases, as well as non-neuroprotective effects in peripheral organs [39–41]. By modulating autophagy, PACAP has beneficial effects on amyotrophic lateral sclerosis, Parkinson’s disease, and liver ischemia–reperfusion injury [42–44]. To our knowledge, no studies have investigated whether PACAP can modulate autophagy in AD.

In this study, we examined whether PACAP is involved in autophagic flux and could have therapeutic effects on AD by means of autophagy. Accordingly, we used an AD mouse model and a mouse hippocampal neuronal cell line (HT22) with oligomeric Aβ42 damage to evaluate changes in autophagy and the therapeutic effects of PACAP. Through an RNA sequencing (RNA-seq) approach, we found that three important pathways were involved: PI3K-AKT, mTOR, and AMPK. Furthermore, Sirt3 knockdown experiments in HT22 cells indicated that Sirt3 is a key component of PACAP-induced effects on autophagy. Furthermore, analyses of human serum samples showed that PACAP modulates autophagy in human cells. These findings suggest that PACAP–Sirt3-mediated changes in autophagy can serve as therapeutic targets for AD.

## Materials and methods

### Intranasal administration of PACAP to mice

Experiments were conducted on C57BL/6 J mice (from Beijing SPF Biotechnology Co., Beijing, China) and 3xTg mice with a background of B6/129 and 3 mutant overexpressed genes (APPSwe, tauP301L, and Psen1tm1Mpm) (from Beijing Vital River Laboratory Animal Technology Co., Ltd, Beijing, China). To avoid the effects of estrogen in female mice, 9-month-old male mice were divided into three groups (*n* = 8–12 per group): wild-type (WT, C57BL/6 J) control, with 0.9% saline, 3xTg (untreated), with 0.9% saline, and 3xTg + PACAP (treated), with PACAP (dissolved in sterile water, 0.1 mg/mL, 6 μL/day, 3 μL per nostril). All three groups received the indicated interventions by intranasal administration (described above) for 4 weeks, as shown in Additional file 1: Supplementary Fig. S1B.

### Behavioral studies

After four weeks of administration, all the mice were tested in behavioral experiments. Morris water maze (MWM) is a behavioral study examining spatial learning and memory [45]. The mice were introduced to a circular pool and then swim freely. The platform was set in northeast quadrant. The time to reach the platform as well as the frequency to cross the platform were recorded in each trial. During the first four days of training, the mice were given 4 trials per day and randomly put into the water in one of the four starting locations with an inter-trial interval of 20 min. When the mouse failed to reach the platform, it would be placed on it for 30 s. While the mouse found the platform successfully, it was permitted to stay for 15 s. On the 5th day, after removing the platform, each mouse was put into southeast quadrant of the pool, and each mouse was observed and recorded in searching of the platform during 120 s. Analysis of the escape latency during training days and frequency to cross the platform position as well as lasting time in target quadrant on the fifth day test were used to examine learning and memory in mice.

Open field trials were used to investigate “anxiety-like” behavior [46]. The mice were placed in an open field, measured 50 cm long, 50 cm wide, and 40 cm high for 10 min. The whole experiment was illuminated by 2 energy-saving lamps hanging from the top, maintaining a sightly dim environment, and keeping quiet throughout. The nine middle squares were seen as the center, the twelve outermost cells were considered peripherals. The distance or time in center and total distance were recorded within 10 min.

After behavioral studies, all mice were euthanized with isoflurane anesthesia, then immediately subjected to intracardially perfusion with saline. The brain of each mouse was carefully removed and divided into two cerebral hemispheres for subsequent analysis.

### RNA–sequencing

Nine-month-aged 3xTg male mice were treated with 0.9% normal saline while age-matched male 3xTg mice were treated with PACAP. After 4 weeks, take out the hippocampus from the mice’s brain and total RNA were extracted using Trizol (Invitrogen, 15596026) following the manual instruction. The following more detailed experimental steps were from MyBGI 3.0 online system (MyBGI 3.0). The significant difference in gene expression between the groups was analyzed under the control of log2 FC > 1, false discovery rate (FDR) q value < 0.05.

### Transmission electron microscopy (TEM)

TEM [47] were used to observe the autophagosome or autolysosome in the mice’s hippocampus, the 5 mm size coronal section of the hippocampus was isolated and soaked with a mixture of 2.5% glutaraldehyde and 2% paraformaldehyde for 24 h. Then the tissue was dehydrated and embedded in the Epon resin. Sections of 70 nm thickness were cut and stained with uranyl acetate and lead citrate. Then randomly selected a region in the hippocampus and calculated the number of autophagosomes or autolysosomes. And to observe the Aβ42 oligomer prepared, we need to use the method of negative staining which in dose not require antibody. Cover the front of the copper net on the drug liquid for 5 min firstly, then blotted with filter paper and placed on the surface of 1% liquid phosphotungstic acid for 5 min to color the drug. After being blotted with a filter paper and baked under incandescent light bulb, until the copper mesh is completely dry, it could be observed under a TEM (H7650, Hitachi, Tokyo, Japan).

### Immunohistochemical staining for Aβ and pTau with light microscopy visualization

Each whole brain was cut in half and the brainstem was discarded. Half of the brain was embedded in paraffin and stored at room temperature. For analysis, embedded brain tissue was sliced into 8-mm sections; the sections were incubated at 60 °C for ≥ 1 h to dissolve paraffin, then hydrated using an alcohol gradient. Subsequently, antigen retrieval was conducted by microwaving in a sodium citrate solution (3 times for 5 min each). Sections were immersed in 0.3% hydrogen peroxide for 10 min in the dark to inactivate peroxidases. Next, they were blocked with 10% goat serum for 1 h at room temperature. Excess serum was removed and sections were incubated with the primary antibodies overnight at 4 °C: mouse anti-pTau (Ser202, Thr205 [AT8]; MN1020, Thermo Fisher Scientific, MA, USA; 1:100 dilution) and mouse anti-Aβ17-24 (800701, BioLegend; 1:500 dilution). Sections were then warmed by incubation at room temperature for ≥ 20 min; they were washed 3 times (5 min each) with phosphate-buffered saline (PBS). Next, sections were incubated at room temperature for 1 h with horseradish peroxidase-linked anti-mouse secondary antibody (8135 s, Cell Signaling Technology; 1:800 dilution). Sections were washed with PBS, developed for 10 min with a diaminobenzidine kit (ZLI-9018, New England Biolabs), and subjected to nuclear staining via hematoxylin. Finally, sections were dehydrated in an alcohol gradient, cleared with xylene, and sealed with neutral resin. They were mounted and observed using an inverted microscope (Vert.A1, Zeiss, Germany).

### Immunofluorescence staining

The detailed methods were described in previous study [38]. Sections were deparaffinized, hydrated, subjected to antigen retrieval, and blocked as described above (no peroxidase inactivation was performed). Sections were incubated with the primary antibody overnight at 4 °C: rabbit anti-LC3A/B (4108 s, Cell Signaling Technology; 1:200 dilution). Sections were washed as described above, then incubated at room temperature for 1 h in the dark with AlexaFluor 647-linked goat anti-rabbit secondary antibody (ab216773, Abcam). Sections were washed with PBS, sealed with 4′,6-diamidino-2-phenylindole (8961, Thermo Fisher Scientific), stored at 4 °C in the dark, and photographed by fluorescence microscopy (Evos FL Auto2, Thermo Fisher Scientific).

### Real-time quantitative polymerase chain reaction (RT-qPCR)

As described before [48], RNeasy Lipid Tissue Mini Kit (74804, Qiagen) was used to extract total RNA from tissues. RNA was then reverse transcribed into cDNA by the Prime Script™ RT Reagent Kit with gDNA Eraser (RR047A, Takara), in accordance with the manufacturer’s protocol. cDNA was amplified by RT-qPCR using TB Green® Premix Ex Taq™ II (RR820A, Takara) with a QuantStudio 3 real-time thermocycler system (Applied Biosystems, USA). Changes in gene expression were analyzed by the 2^−ΔΔCt^ method and normalized to glyceraldehyde-3-phosphate dehydrogenase (GAPDH). Primer sequences are provided in Additional file 1: Supplementary Table S1.

### Preparation of oligomeric Aβ42

Human synthetic Aβ42 (rPeptide, A-1163–2) was dissolved in 222 μl hexafluoroisopropanol and conFig.d to 1 mmol/l, then ultrasonicated over ice for 3 times, 5 s per time. After standing at room temperature for 60 min, put it in a hood to evaporate overnight until it forms a transparent film (oligomeric Aβ42). Then it was preserved at -80 °C. Took it out of the fridge, treated it in cultural media, and incubated it at 4 °C for 24 h prior to use. It could be verified by TEM (Fig. 4A) or Western blot [45, 49], and it is almost same gel as described in western blot section.

### Cell culture and the treatment with Aβ42 oligomer and PACAP1-38

HT22 was a mouse hippocampal neuronal cell line. It was cultured in Dulbecco's Modified Eagle Medium (Gibco, 11960044; DMEM), added by 10% fetal bovine serum and 0.01% pen strep glutamine (Life, 10378016). Cells were incubated in the humidified condition of 37 °C with 5% CO_2_, 94% N_2_, and 1% O_2_, treated with 20 μM Aβ42 oligomer with or without 100 nM PACAP-38 for 24 h, reported in previous study [38]. PACAP-38 was dissolved in sterile water with a density of 0.1 mg/ml and diluted freshly to 100 nM in the culture medium.

### Cell counting kit-8 (CCK-8) for cell viability assay

CCK-8 solution can be directly added to cell samples to determine the number of living cells in cell proliferation or toxicity tests. It is a non-radioactive colorimetric test. The HT22 cellular viability was tested by CCK-8 assay. The treated cells were seeded into 96 well plates (10000 cells per well) and incubated with CCK-8 for 50 min according to cell proliferation, then the viability (OD) was assessed under 450 nm absorbance (DOJINDO, CK04; Kyushu, Japanese).

### Serum and Glucose Deprivation (SGD)

The HT22 cells were seeded into 6 wells plate with Dulbecco's Modified Eagle Medium (DMEM) for 24 h. On the second day, the medium was discarded and cells were washed with 1 × PBS, and changed the medium to SGD buffer for 12 h.

### LC3 turnover assay

This experiment was used to determine whether the reduction in autophagy was due to the blocked fusion of autophagosome and lysosome. Chloroquine (CQ), as an autophagy blocker, decreases autophagosome-lysosome fusion, inhibits the degradation of LC3-II, results in the accumulation of LC3-II and inhibits autophagic flux [3, 50]. We used CQ 20 μM for 24 h to inhibit autophagy with increased LC3-II:I ratio. There were 4 groups of the cells: cells without any treatment; cells with CQ; cells with an intervention needed for the experiment; cells with intervention and CQ.

### Sirt3 vector construction and transfection

To inhibit Sirt3 on HT22 cell, the sequence of 19 nucleotides was constructed, which targeted Sirt3 location 764 into Omics Link small hairpin RNA (shRNA) expression clone. And the Sirt3 shRNA vector and NC control vector were packaged into the Lentivirus transfection system. Because the vector has EGFP, to confirm the transfection efficiency, the fluorescence of transfected cells could be observed, and could be tested by western blot.

### Flow cytometry analysis

Before flow cytometry, HT22 cells were transfected with mRFP-GFP-MAP1LC3B (Genechem). When autolysosomes are formed, GFP is quenched by the acidic environment; thus, phycoerythrin-area (PE-A, overlapping RFP and GFP signals) represents autolysosomes, whereas peridinin-chlorophyll-protein complex-area (PerCP-A, RFP signal alone) represents autophagosomes. Treated cells were washed with PBS for three times and suspended in 500 μl PBS, then filtered with 300-mesh nylon and subjected to flow cytometry for spectral analysis.

### Western blotting

The detailed methods were described in previous study [38]. Brain tissue and treated cells were homogenized in cold cell lysis buffer (9803S, Cell Signaling Technology) containing protease inhibitors and phosphatase inhibitors, then subjected to protein extraction. Protein samples were heated at 95 °C for 5 min, then mixed with loading buffer at a 1:1 ratio. Samples were subjected to electrophoresis on 8% or 12% gels (SDS-PAGE Gel Kit, P1200, Slaribio; 30 μg total protein per well) and transferred to a nitrocellulose membrane (66,485, BioTrace) at 4 °C for approximately 1–2 h (transfer time was dependent on protein weight). Subsequently, the membrane was incubated at room temperature for 1 h with 5% milk, then incubated overnight at 4 °C with primary antibody (listed in Additional file 1: Supplementary Table S2). The membrane was washed four times (5 min each) with Tris-buffered saline plus 0.1% Tween (TBST, T1085, Slaribio), then incubated at room temperature for 1 h with secondary antibody (listed in Additional file 1: Supplementary Table S2). Finally, the membrane was washed with TBST buffer and protein signals were quantified using an Odyssey Fc device (LI-COR Biosciences, Lincoln, NE, USA).

### Study design and participants

China Alzheimer's Disease and Neurodegenerative Disorder Research (CANDOR) is a prospective cohort study [51]. This study is a sub-study of CANDOR, began in July 2019 and ended in July 2021; participants consisted of patients with cognitively normal and AD individuals. Diagnostic criteria for AD were met according to the 2011 guidelines of the National Institute on Aging and Alzheimer's Association (NIA-AA) [52]. All participants completed Pittsburgh compound B positron emission tomography–computed tomography. Fasting peripheral blood was collected from each participant for the detection of AD-related biological markers, and all participants completed the Clinical Dementia Rating (CDR) Global Score assessment. ApoE genotype data are provided in Additional file 1: Supplementary Table S3.

### Isolation of neuronal-derived exosomes from human serum for western blot and enzyme-linked immunosorbent assay (ELISA)

The protocol was described before [53], serum was collected and centrifuged at 3,000 × g for 15 min to remove cell debris, then the whole exosome precipitation was prepared according to the instructions of ExoQuick (Systembio, EQULTRA-20A-1), and dissolved by Dulbecco's phosphate-buffered saline (Gibco, 14190). Then the neuronal-derived exosomes were enriched with biotin-labeled anti-human NCAM antibodies (Santa Cruz Biotechnology, sc-106). Finally, PACAP (Santa Cruz Biotechnology, SC-166180) and MAP1LC3B (Fine Test, EH2243) concentrations were fully quantified by ELISA kits according to the instructions, and LC3 (Cell Signaling Technology, 4108S) was semi-qualified by western blot simultaneously.

### Measurements of Aβ42, pTau, and total Tau in plasma

The collected blood was centrifuged to remove cell debris, then the Aβ42, pTau, and total Tau were determined by the Simoa kits (Quanterix, 103714, 101195). And we performed the tests by HD-X analyzer (Quanterix), according to the manual [54].

### Statistical analysis

Results are presented as mean ± standard deviations. We perform the analysis through GraphPad Prism 6 and IBM SPSS Statistics 23.0 software. The chi-squared test was used to analyze categorical variables. Normality was determined by the Shapiro–Wilk test. Differences between groups were analyzed by the Mann–Whitney U test (non-normally distributed data) or two-tailed unpaired Student’s t-tests (normally distributed data). One-way analysis of variance was used for comparisons of ≥ 3 groups. Linear correlation analysis was used to calculate pairwise relationships between variables. Linear regression analysis was performed to evaluate the association between AD and MAP1LC3B, PACAP of different models (Model 1: age; Model 2: age and sex. Beta values and their 95% confidence intervals (CI) were calculated separately. Values of *p* < 0.05 were considered statistically significant; in all analyses, mice and cells were randomly divided into different groups. All experiments were repeated at least three times.

## Results

### PACAP improved cognitive function in 3xTg mice

**Fig. 1:**
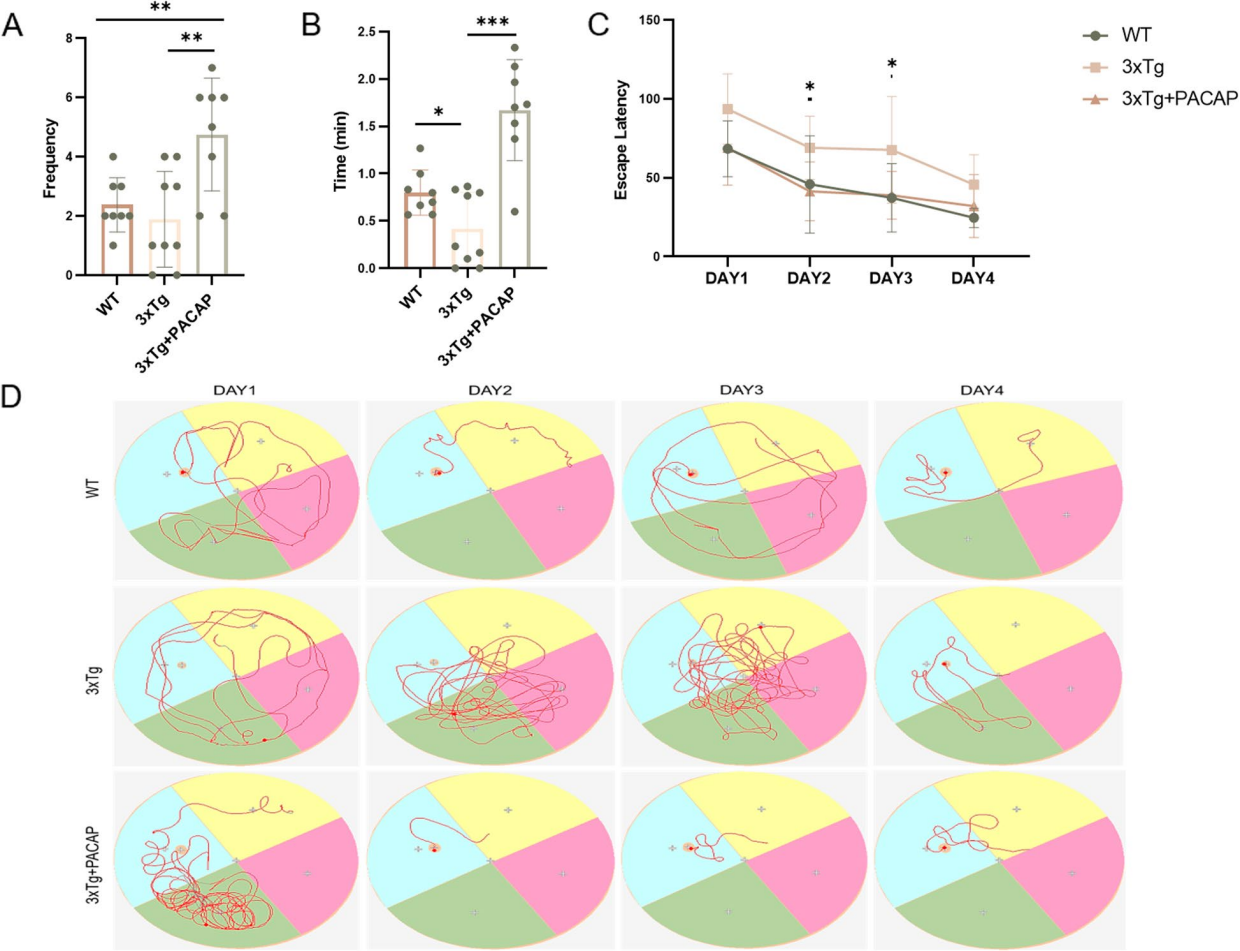
Behavioral experiments showed that cognitive impairment in 3xTg mice was alleviated by PACAP. **A** Water maze frequency of platform crossing, compared among WT, 3xTg, and 3xTg + PACAP groups. WT = wild-type mice treated with 0.9% normal saline, 3xTg = APP/PS1/tau triple-transgenic mice treated with 0.9% normal saline, and 3xTg + PACAP = APP/PS1/tau triple-transgenic mice treated with PACAP (0.6 μg per day for 4 weeks). **B** Total times mice spent in the quadrant where the platform had been located. **C** Escape from the water maze to the platform within the first 4 days of training. **D** Movement trajectories of mice on the first 4 days of water maze training. *n* = 8–9. *p < 0.05, **p < 0.01, ***p < 0.001

After 4 weeks of PACAP or normal saline treatment, all three groups of mice underwent MWM testing to assess their learning and memory performances. The three groups showed significant differences in platform crossing frequency (*p* = 0.002, F(2, 22) = 8.144; Fig. 1A). Compared with 3xTg untreated mice, wild-type (WT) control mice spent 91.28% more time in the target quadrant where the platform had been placed (*p* = 0.028; Fig. 1B). Compared with 3xTg untreated mice, 3xTg + PACAP mice passed the platform more frequently (*p* = 0.004; Fig. 1A) and spent 299.76% more time in the target quadrant (*p* < 0.001; Fig. 1B). The results indicated that PACAP improved memory performance in 3xTg mice. In terms of escape latency (i.e., time for mice to initially find the platform), escape latency on the second day was shorter in 3xTg + PACAP mice than in 3xTg untreated mice; thus, 3xTg + PACAP mice had better learning performance (*p* < 0.05; Fig. 1C, D). Additionally, mice were subjected to open field trials for assessment of anxiety. The results showed that 3xTg untreated mice explored less and stayed in the center for a shorter time, compared with WT; 3xTg + PACAP mice did not exhibit these tendencies (*p* < 0.05; Additional file 1: Supplementary Fig. S2B and C). Total distance traveled by 3xTg untreated mice was shorter than the distance traveled by WT mice (*p* < 0.05); however, the distance traveled by 3xTg + PACAP mice did not differ from the distance traveled by 3xTg untreated mice (*p* = 0.611; Additional file 1: Supplementary Fig. S2A).

### Autophagy-related mRNA profiles were altered in PACAP-treated 3xTg mice

**Fig. 2:**
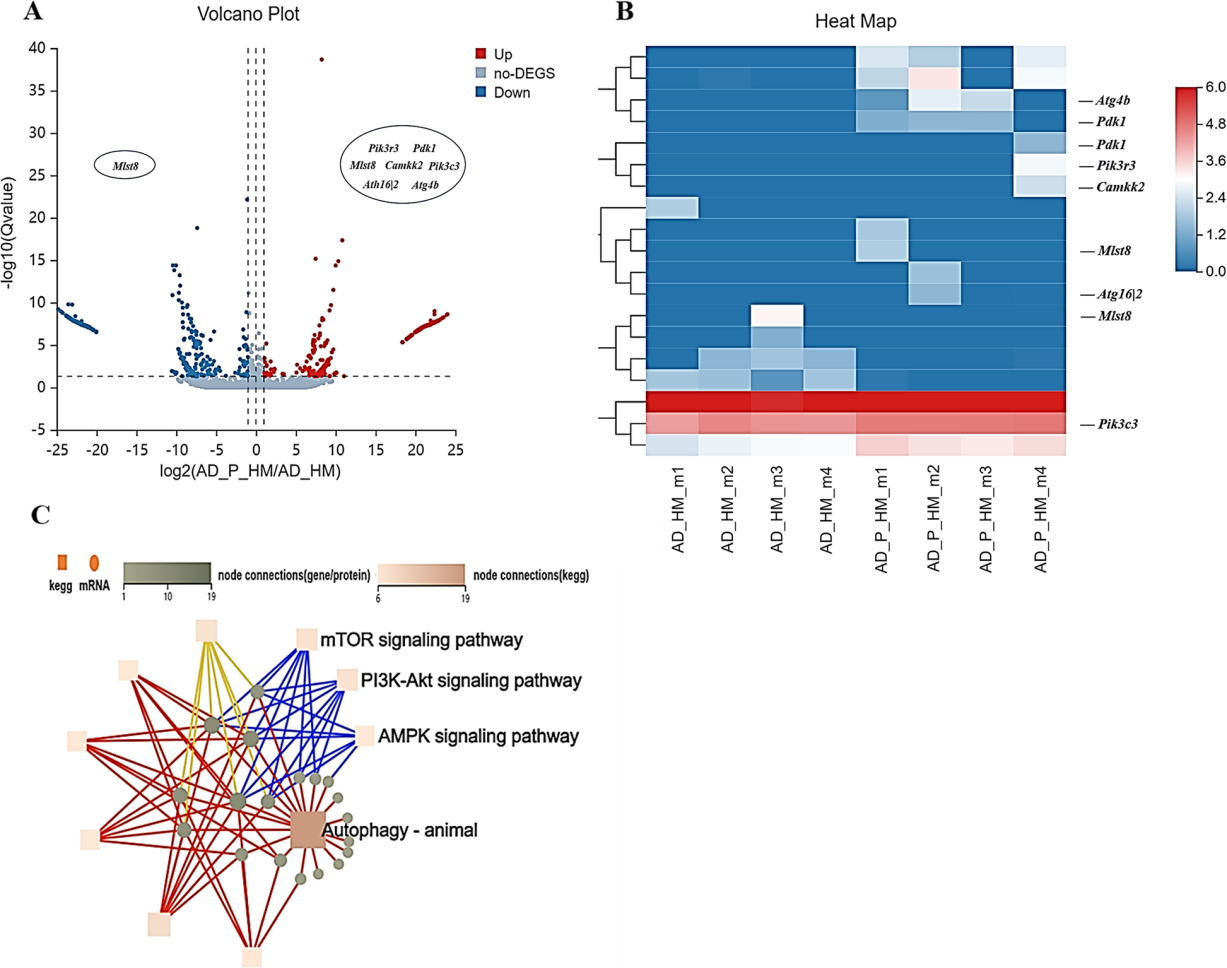
mRNA profiling showed that PACAP altered the expression of autophagy-related genes in 3xTg mice. **A** Volcano plot shows 614 upregulated genes and 643 downregulated genes in PACAP-treated APP/PS1/tau triple-transgenic mice compared with normal saline-treated APP/PS1/tau triple-transgenic mice. **B** Heat map shows differentially expressed genes in PACAP-treated AD model mice. **C** KEGG pathway networks show several important autophagy-related pathways. *n* = 4

To study the effects of PACAP on autophagy, we performed RNA-seq on 3xTg mice treated or not with PACAP (0.6 μg/day for 4 weeks). Mouse hippocampal mRNA profiles were filtered with SOAPnuke (v1.5.2) [55], and differentially expressed genes were analyzed using the Dr. Tom online platform (BGI Genomics Co., Ltd.). A volcano plot and heat map were generated from the mRNA profiles (Fig. 2A, B). There were 614 upregulated mRNAs and 643 downregulated mRNAs; 19 mRNAs were related to autophagy (log2 fold change > 1, false discovery rate < 0.05). Among these 19 mRNAs, the autophagy stimulators Ca^2+^/calmodulin-dependent protein kinase-β (a molecule upstream of AMPK), VPS34 (Pik3c3, a Beclin1 binding partner [24, 56]), ATG16 (Atg16l2, a subunit of the ATG12-ATG5-ATG16 complex [57, 58]) and ATG4 (Atg4b) were all increased. The mTOR-associated protein MLST8 (a component of mTOR [59]) has multiple transcripts: NM_001252463.1 was decreased, whereas XM_006524679.5 was increased. Transcripts of total PI3K (Pik3r3) and pyruvate dehydrogenase kinase 1 (PDK1, which promotes phosphorylation of AKT [60]) were increased. Importantly, this method did not provide data concerning the expression of total AKT and phosphorylated PI3K, or the ratios of p-PI3K:PI3K and phosphorylated AKT (p-AKT):AKT.

Kyoto Encyclopedia of Genes Genomes (KEGG) and Gene Ontology (GO) enrichment analyses revealed several KEGG pathways associated with autophagy (Fig. 2C). The top 20 GO and KEGG terms are shown in Supplementary Additional file 1: Fig. S3A. Three other KEGG pathways for neurodegenerative diseases (amyotrophic lateral sclerosis, spinocerebellar ataxia, and Parkinson’s disease) were identified by gene set enrichment analysis, as shown in Additional file 1: Supplementary Fig. S3B. Overall, the RNA-seq results suggested that PACAP treatment has an important effect on autophagy in 3xTg mice.

### PACAP restored autophagy function in vivo and in vitro

**Fig. 3:**
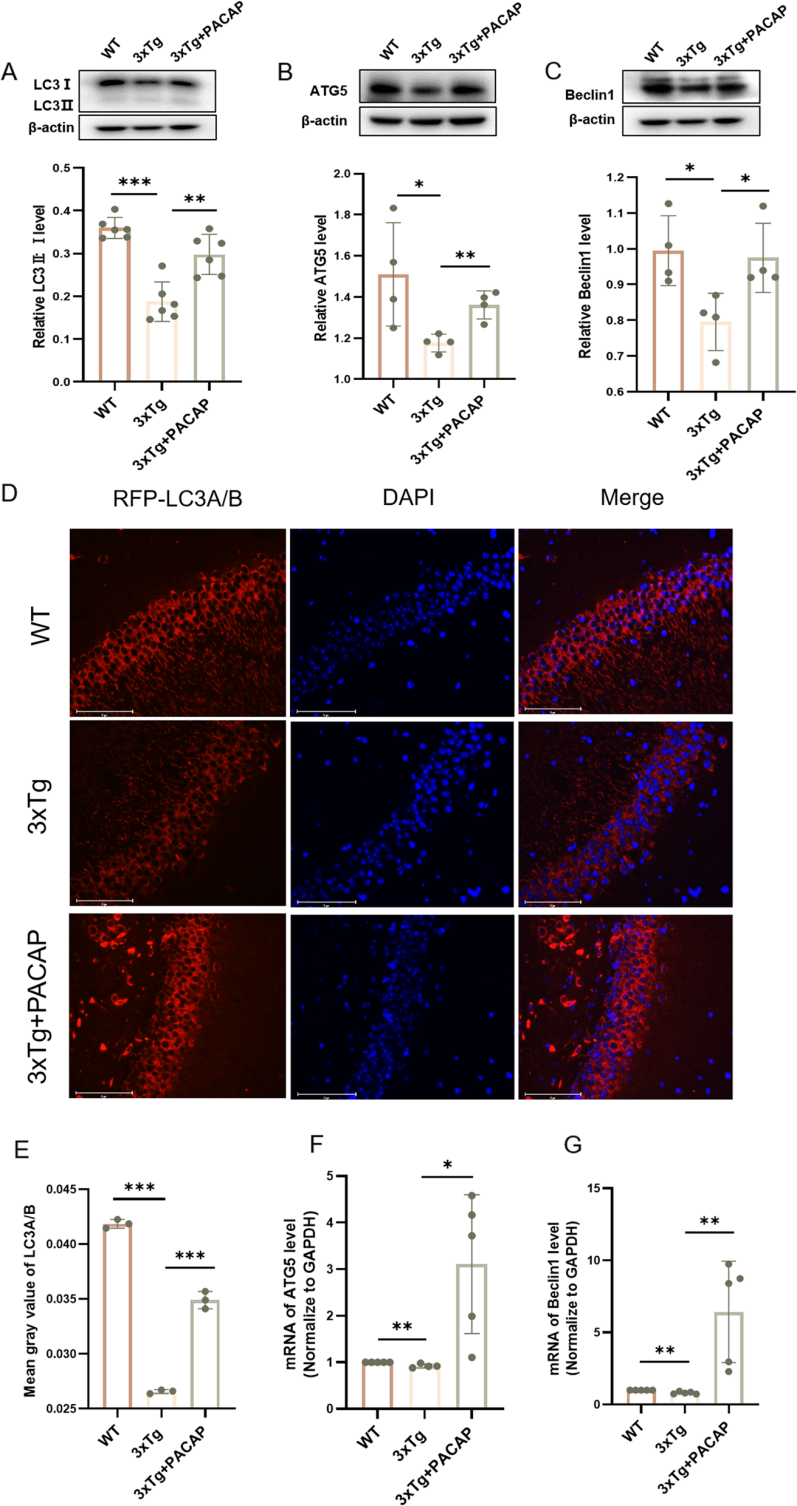
Expression levels of autophagy biomarkers in 3xTg mice were increased by PACAP treatment. Western blots and corresponding graphs show hippocampal protein levels of (**A**) LC3-II:I (*n* = 6), (**B**) ATG5 (*n* = 4), and (**C**) Beclin1 (*n* = 4). **D** Immunofluorescence staining of LC3A/B in the hippocampus of WT, 3xTg, and 3xTg + PACAP mice. Red color (RFP) represents LC3A/B; blue color (DAPI) represents cell nuclei. Scale bar, 75 μm; *n* = 3. **E** Quantitative analysis of MAP1LC3A/B fluorescence density. RT-qPCR analysis of mRNA levels of (**F**) ATG5 and (**G**) Beclin1 in the cortex of 3xTg mice, *n* = 4–5. *p < 0.05, **p < 0.01, ***p < 0.001

Autophagy proteins in WT, 3xTg untreated, and 3xTg + PACAP mice were measured by western blotting. The LC3-II:I ratio, ATG5 level, and Beclin1 level were lower in 3xTg untreated mice than in WT mice (*p* < 0.001, *p* = 0.039, and *p* < 0.019, respectively); these levels were higher in 3xTg + PACAP mice (3xTg + PACAP vs. 3xTg untreated, all *p* < 0.05; Fig. 3A, B, C). Immunofluorescence staining of LC3A/B in the mouse hippocampus showed similar results (*p* < 0.001; Fig. 3D, E). RT-qPCR proved that the mRNA levels of ATG5 and Beclin1 were similar to the protein levels in western blotting (both *p* < 0.05; Fig. 3F, G).

**Fig. 4:**
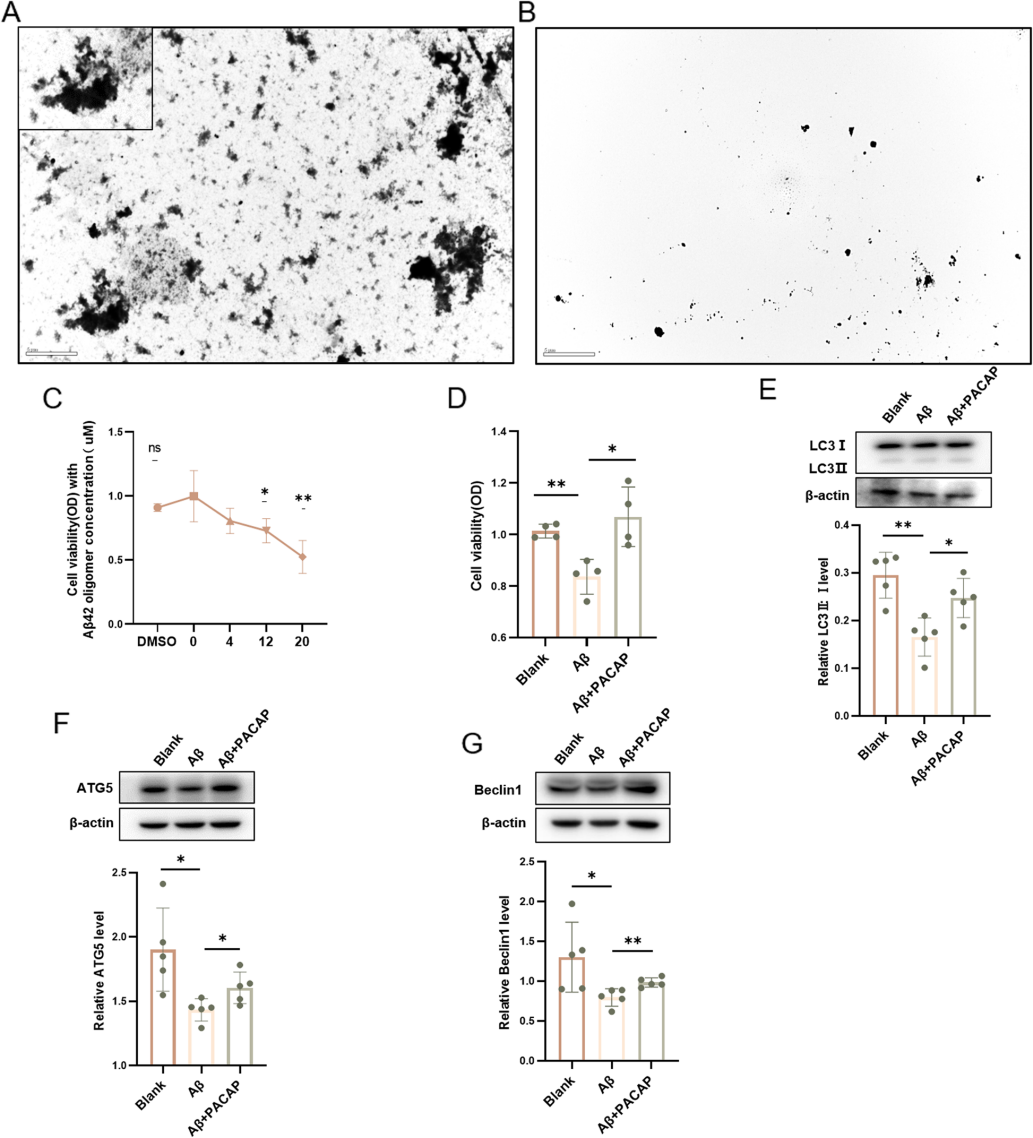
The expression of autophagy biomarkers in HT22 cells was increased after PACAP treatment. **A** Electron microscope image of oligomeric Aβ42; scale bar, 5 μm. **B** Electron microscope image of culture media without oligomeric Aβ42; scale bar, 5 μm. **C** HT22 cell viability (measured by optical density [OD]) after treatment with various concentrations of oligomeric Aβ; *n* = 4–6. **D** HT22 cell viability after no treatment (control group), oligomeric Aβ42 (20 μM) treatment, and oligomeric Aβ (20 μM) + PACAP (100 nM) treatment; *n* = 4. Western blots and corresponding graphs show protein levels of (**E**) LC3-II:I, (F) ATG5, and (**G**) Beclin1 in HT22 cells after no treatment (control group), oligomeric Aβ42 treatment for 24 h (Aβ group) and oligomeric Aβ42 + PACAP treatment for 24 h (Aβ + PACAP group), *n* = 5. *p < 0.05, **p < 0.01

To investigate effects on autophagy in vitro, HT22 cells were divided into three groups: blank control (no treatment), Aβ (treatment with oligomeric Aβ42 for 24 h), and Aβ + PACAP (treatment with oligomeric Aβ42 and PACAP for 24 h). The preparation of oligomeric Aβ42 was confirmed by electron microscopy (Fig. 4A, B).

Treatment with 12 μM and 20 μM oligomeric Aβ42 reduced HT22 cell viability by 27.03% and 47.46%, respectively (*p* < 0.05); in cells that were also treated with PACAP, viability remained near 100% (*p* = 0.013; Fig. 4C, D). Treatment with 20 μM oligomeric Aβ42 reduced the LC3-II:I ratio (21.73% decrease vs. 0 μM, *p* = 0.002; Fig. 4E), ATG5 level (24.62% decrease vs. 0 μM, *p* = 0.014; Fig. 4F), and Beclin1 level (38.88% decrease vs. 0 μM, *p* = 0.037; Fig. 4G). Concurrent treatment with 100 nM PACAP led to a 49.37% higher LC3-II:I ratio, 11.93% higher ATG5 level, and 23.68% higher Beclin1 level (20 μM oligomeric Aβ42 + 100 nM PACAP vs. 20 μM oligomeric Aβ42 alone, *p* < 0.05; Fig. 4E, F, G).

**Fig. 5:**
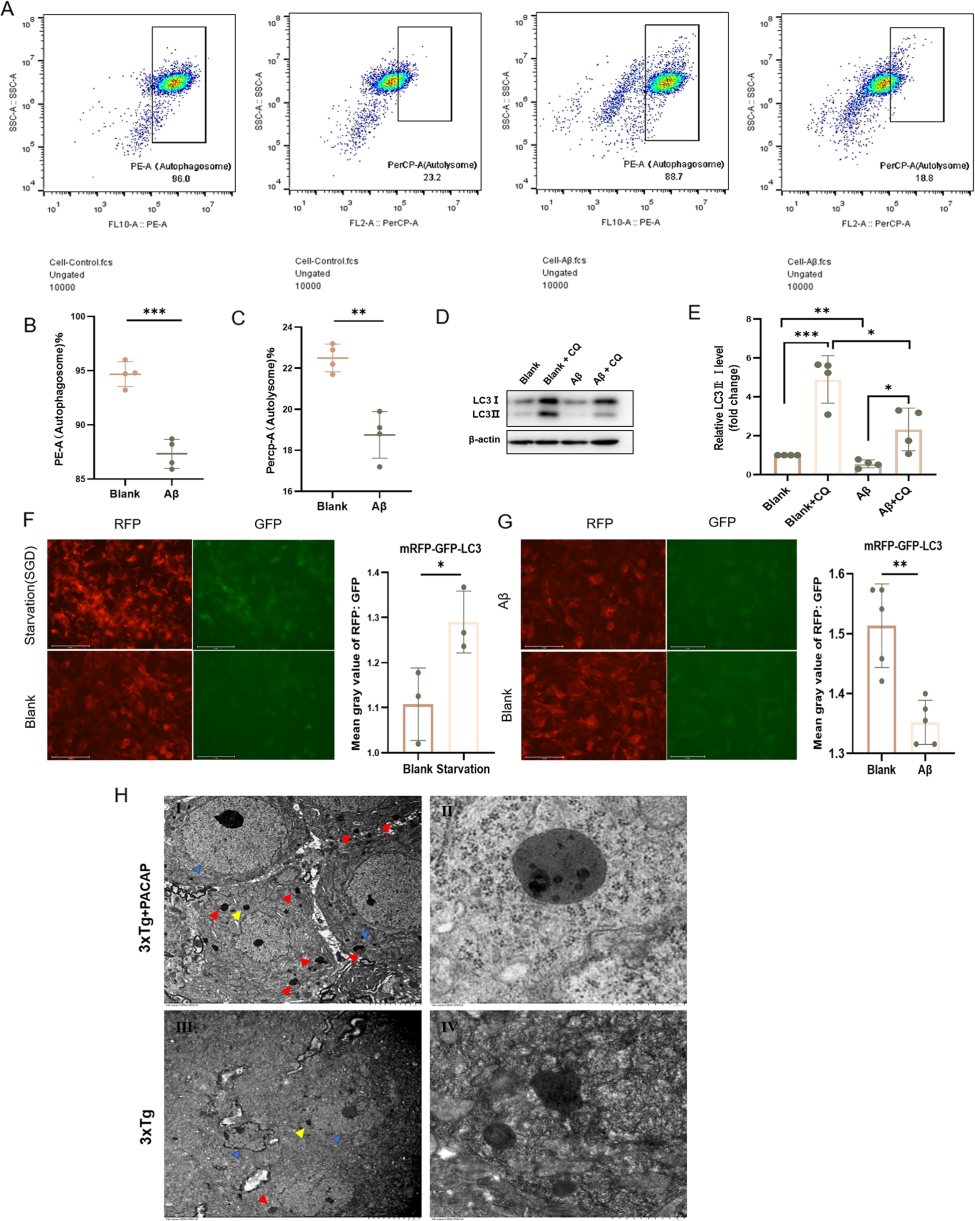
Additional experiments showed autophagy impairment in an in vitro model of AD. Flow cytometry was used to detect (**A**) autophagic flux in mRFP-GFP-LC3 HT22 cells with or without oligomeric Aβ42 damage for 24 h; corresponding graphs show changes in (**B**) autophagosomes and (**C**) autolysosomes; *n* = 4. **D** Western blots and (**E**) corresponding graphs show protein levels of LC3-II:I with in the control, control + CQ for 24 h, Aβ (oligomeric Aβ42 intervention for 24 h), and Aβ + CQ (oligomeric Aβ42 and CQ treatment for 24 h) groups; *n* = 4. **F** mRFP-GFP-LC3 HT22 cell fluorescence in the control and starvation (serum and glucose deprivation [SGD]) groups; corresponding graph shows RFP:GFP ratio. Scale bar, 75 μm; *n* = 3. **G** mRFP-GFP-LC3 HT22 cell fluorescence in control and Aβ groups; corresponding graph shows RFP:GFP ratio. Scale bar, 75 μm; *n* = 5. **H** Transmission electron microscope images of the hippocampus of 3xTg and 3xTg + PACAP mice: (i and iii) morphology of nuclei (blue arrows) and autophagosomes or autolysosomes (red and yellow arrows); scale bar, 5 μm. (ii and iv) Enlarged depictions of (i and iii) (yellow arrows); scale bar, 0.5 μm. *p < 0.05, **p < 0.01, ***p < 0.001

Flow cytometry (Fig. 5A) showed that treatment with 20 μM oligomeric Aβ42 reduced both autophagosome formation (7.76% decrease vs. 0 μM, *p* < 0.001; Fig. 5B) and autolysosome formation (16.67% decrease vs. 0 μM, *p* = 0.001; Fig. 5C). The LC3-II:I ratio was also decreased after treatment with oligomeric Aβ42 and chloroquine (CQ), compared with control treatment plus CQ (53.53% decrease vs. control, *p* = 0.02; Fig. 5D, E). Treatment with oligomeric Aβ42 reduced the RFP:GFP ratio by 10.64% (*p* = 0.002; Fig. 5G), whereas starvation (i.e., serum and glucose deprivation, an activator of autophagy) increased it (16.43% increase with serum and glucose deprivation for 12 h vs. 0 h, *p* = 0.04; Fig. 5F).

TEM was used to observe autophagosome or autolysosome formation in the nuclei of hippocampal neurons. The 3xTg untreated mice showed nuclear atypia and diminished autophagic flux (Fig. 5Hiii, iv); the 3xTg + PACAP mice had normal nuclear morphology, intact nuclear membranes, and comparatively more autophagosomes and autolysosomes (relative to 3xTg untreated mice; Fig. 5Hi, ii).

### Amounts of Aβ, total Tau, and pTau deposition were reduced by PACAP treatment in vivo and in vitro

**Fig. 6:**
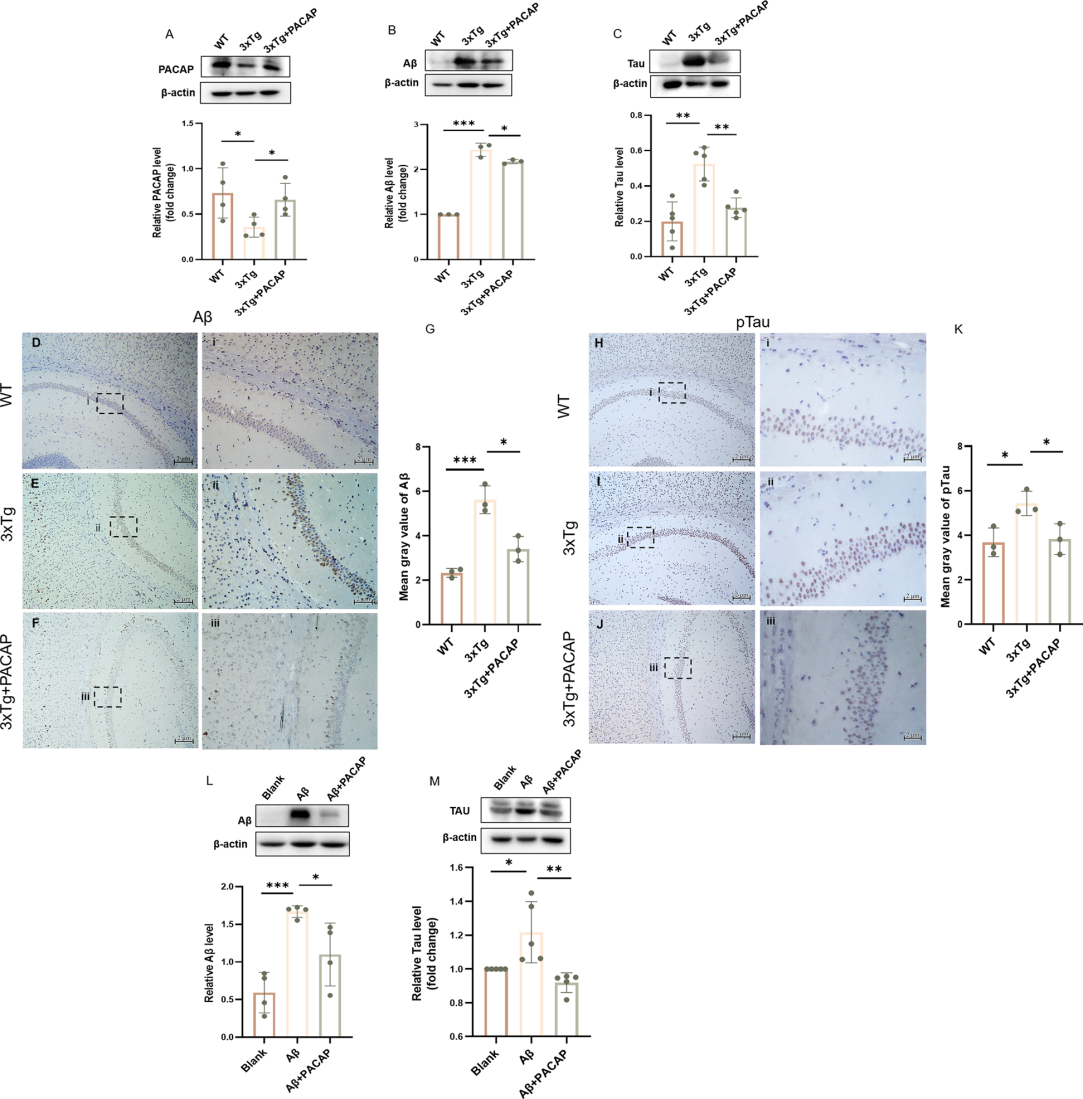
Pathological protein accumulation was alleviated by PACAP treatment in vivo and in vitro. Western blots and corresponding graphs show protein levels of (**A**) PACAP (*n* = 4), (**B**) Aβ (*n* = 3), and (**C**) total Tau (*n* = 5). Immunohistochemical staining of Aβ in the hippocampus of WT (**D** and Di), 3xTg (**E** and Eii), and 3xTg + PACAP mice (**F** and Fiii). Scale bar, 2 μm. **G** Quantitative analysis of Aβ density (*n* = 3). Immunohistochemical staining of pTau in the hippocampus of WT (**H** and Hi), 3xTg (**I** and Iii), and 3xTg + PACAP mice (**J** and Jiii). Scale bar, 2 μm. **K** Quantitative analysis of pTau fluorescence density (*n* = 3). Western blots and corresponding graphs show protein levels of (**L**) Aβ (*n* = 4) and (**M**) total Tau in HT22 cells, *n* = 5. *p < 0.05, **p < 0.01, ***p < 0.001

Hippocampal PACAP levels were lower in 3xTg untreated mice than in WT mice (51.34% decrease vs. WT, *p* = 0.044; Fig. 6A). In 3xTg + PACAP mice, PACAP levels were increased by 45.84% (3xTg + 100 nM PACAP vs. 3xTg untreated, *p* = 0.029; Fig. 6A). In 3xTg untreated mice, Aβ and total Tau deposition were observed; in 3xTg + PACAP mice, the amounts of accumulation were 10.79% and 47.13% lower, respectively (*p* < 0.05; Fig. 6B, C). Immunohistochemistry of hippocampal tissue showed similar results: the amount of Aβ deposition was 39.53% lower in 3xTg + PACAP mice (*p* = 0.011; Fig. 6D, E, F, G), whereas the amount of pTau deposition was 29.58% lower (*p* = 0.034; Fig. 6H, I, J, K).

In HT22 cells treated with oligomeric Aβ42 for 24 h, Aβ deposition was increased by 181.64% (increase vs. 0 μM Aβ, *p* = 0.0003; Fig. 6L); Tau deposition was also increased (21.70% increase vs. 0 μM Aβ, *p* = 0.028; Fig. 6M). In cells treated with both oligomeric Aβ42 and PACAP, Aβ deposition was 34.15% lower than in cells treated with oligomeric Aβ42 alone (*p* = 0.037; Fig. 6L); Tau deposition was reduced by 24.50% (*p* = 0.008; Fig. 6M).

### Three pathways were altered by PACAP treatment in vivo and in vitro

**Fig. 7:**
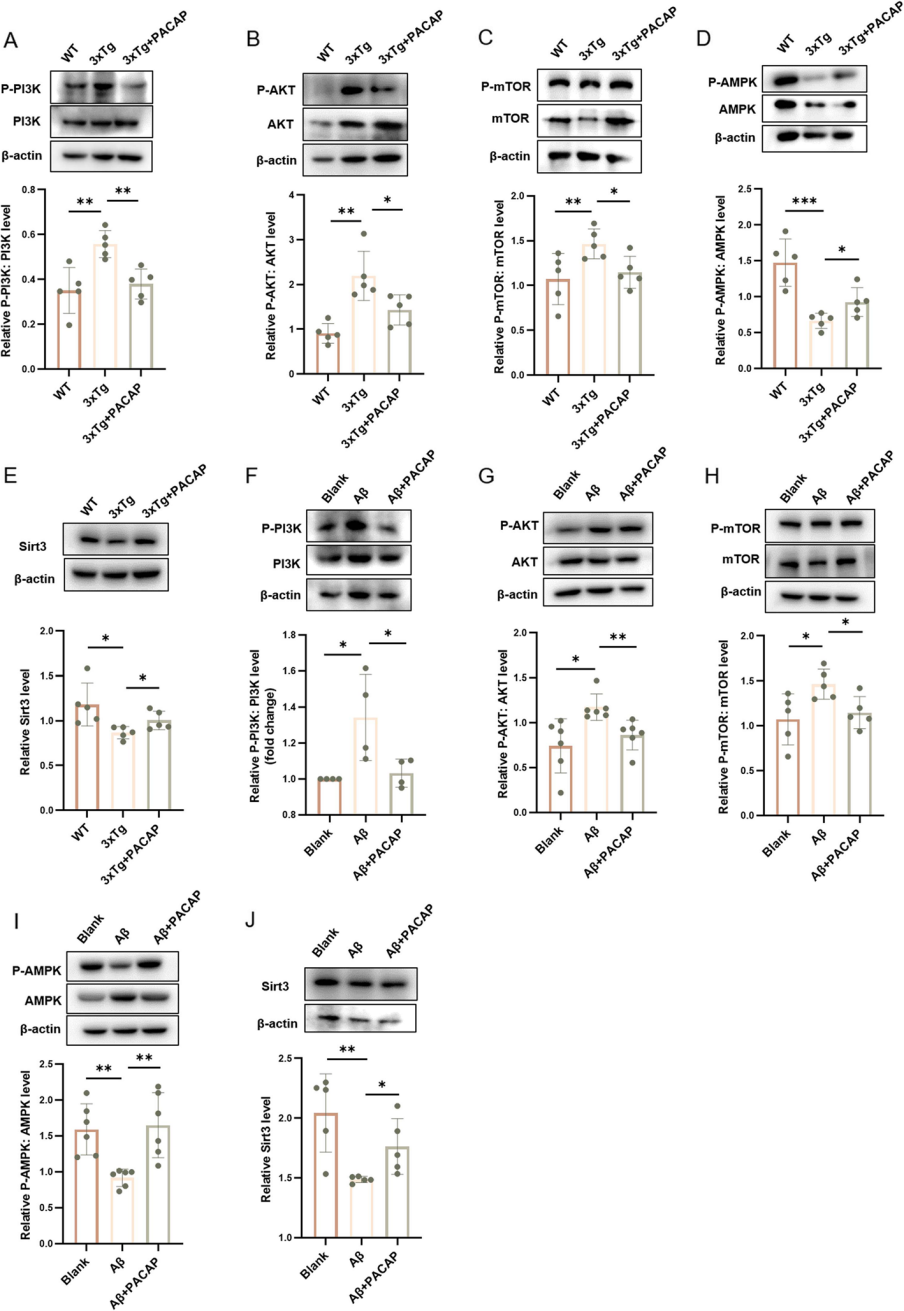
Three autophagy pathways were affected by PACAP treatment in 3xTg mice and HT22 cells. Western blots and corresponding graphs show protein levels of (**A**) p-PI3K:PI3K ratio (*n* = 5), (**B**) p-AKT:AKT ratio (*n* = 5), (**C**) p-mTOR:mTOR ratio (*n* = 5), (**D**) p-AMPK:AMPK ratio (*n* = 5), and (**E**) Sirt3 (*n* = 5) in mouse hippocampus. Western blots and corresponding graphs show protein levels of (**F**) p-PI3K:PI3K ratio (*n* = 4), (**G**) p-AKT:AKT ratio (*n* = 6), (**H**) p-mTOR:mTOR ratio (*n* = 5), (**I**) p-AMPK:AMPK ratio (*n* = 6), and (**J**) Sirt3 (*n* = 5) in HT22 cells, *n* = 5. *p < 0.05, **p < 0.01, ***p < 0.001

To determine whether the PI3K-AKT, mTOR, or AMPK pathways were involved in the effects of PACAP, we tested the following protein expression ratios in WT control, 3xTg untreated and 3xTg + PACAP mice: p-PI3K:PI3K, p-AKT:AKT, phosphorylated mTOR (p-mTOR):mTOR, and phosphorylated AMPK (p-AMPK):AMPK. The p-PI3K:PI3K, p-AKT:AKT, and p-mTOR:mTOR ratios were increased by 59.01% (*p* = 0.005; Fig. 7A), 140.91% (*p* = 0.001; Fig. 7B), and 132.02% (*p* = 0.035; Fig. 7C), respectively, in the 3xTg untreated group compared with the WT control group. Compared with 3xTg untreated mice, 3xTg + PACAP mice exhibited lower p-PI3K:PI3K, p-AKT:AKT, and p-mTOR:mTOR ratios (*p* < 0.05; Fig. 7A, B, C). The P-AMPK:AMPK ratio in the 3xTg untreated group was 55.02% lower than the level in the WT control group (*p* = 0.0008) and 39.52% lower than the level in the 3xTg + PACAP group (*p* = 0.033; Fig. 7D). These findings were consistent with the results of RNA-seq analysis.

In vitro assays showed that autophagy in HT22 cells was restored by PACAP through inhibition of the PI3K-AKT pathway (20 μM Aβ + 100 nM PACAP vs. 20 μM Aβ alone, *p* < 0.05; Fig. 7F, G) and mTOR pathway (20 μM Aβ + 100 nM PACAP vs. 20 μM Aβ alone, *p* = 0.019; Fig. 7H), along with activation of the AMPK pathway (20 μM Aβ + 100 nM PACAP vs. 20 μM Aβ alone, *p* = 0.003; Fig. 7I). Therefore, these three pathways may be involved in the effects of PACAP on pathophysiology in 3xTg mice.

Because Sirt3 plays a pivotal role in neuronal metabolism, we measured it by western blotting. The expression of Sirt3 decreased in the 3xTg untreated group (26.73% decrease vs. WT control, *p* = 0.022) and partially restored in the 3xTg + PACAP group (16.06% increase vs. 3xTg untreated, *p* = 0.037; Fig. 7E). Similar changes in Sirt3 expression were observed in vivo (*p* < 0.05; Fig. 7J). These results exhibit that PACAP can restore Sirt3 expression in vivo and in vitro.

### Sirt3 is a key protein that regulates autophagy in a cellular model of AD

**Fig. 8:**
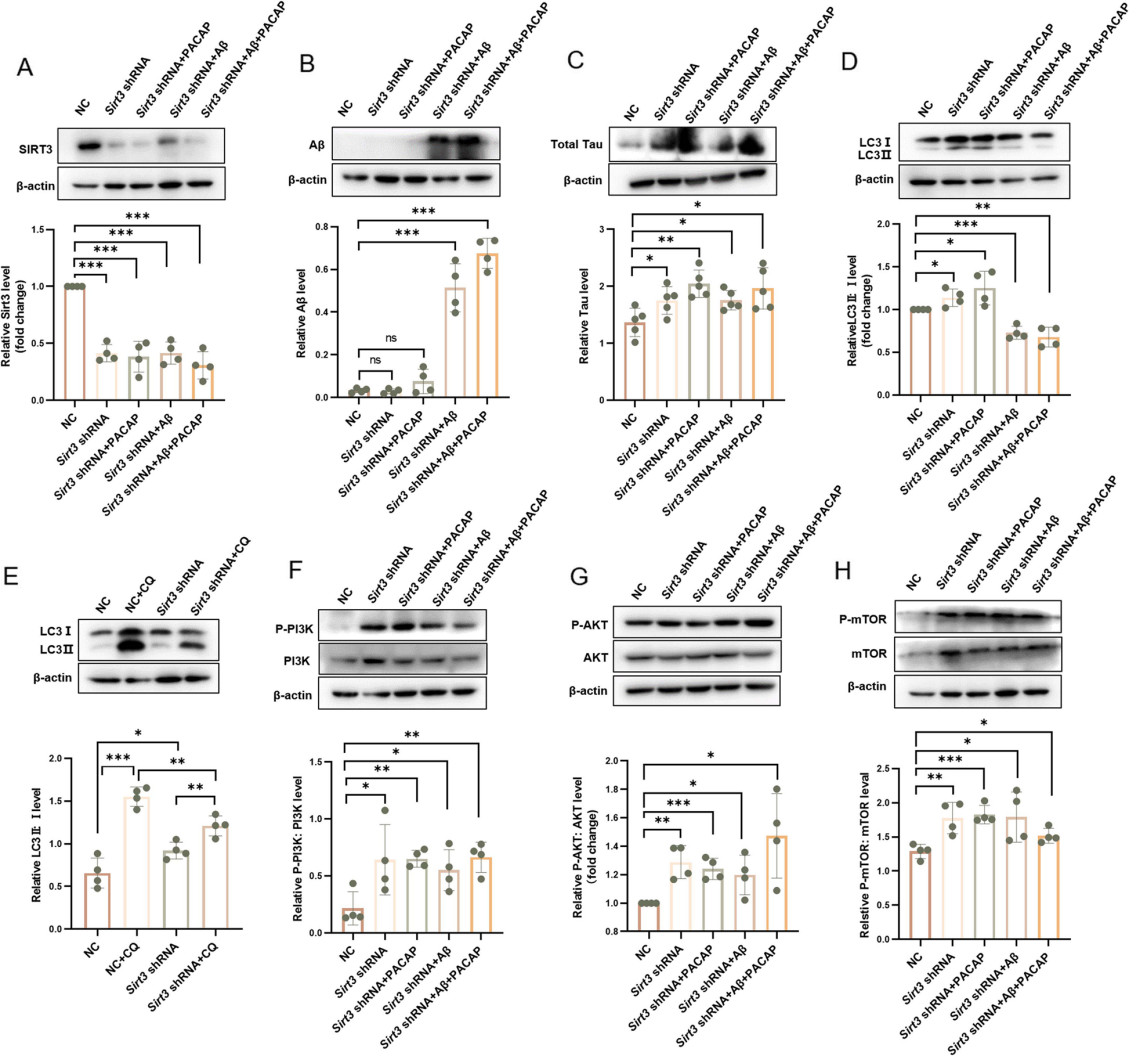
Sirt3 knockdown abolished the effects of PACAP and reduced autophagy in HT22 cells. **A** Western blots and corresponding graphs show protein levels of Sirt3 after Sirt3 knockdown; *n* = 4. Western blots and corresponding graphs show protein levels of (**B**) Aβ (*n* = 4) and (**C**) total Tau (*n* = 5) after Sirt3 knockdown. Western blots and corresponding graphs show protein levels of (**D** and **E**) LC3-II:I, (**F**) p-PI3K:PI3K ratio, (**G**) p-AKT:AKT ratio, and (**H**) p-mTOR:mTOR ratio in the following treatment conditions: NC, Sirt3 knockdown, Sirt3 knockdown + PACAP treatment, Sirt3 knockdown + oligomeric Aβ exposure, and Sirt3 knockdown + oligomeric Aβ exposure + PACAP treatment, *n* = 4. *p < 0.05, **p < 0.01, ***p < 0.001

Sirt3 reportedly attenuates Aβ-induced neuronal hypometabolism and alleviates memory loss [61]. To determine whether PACAP enhanced autophagy via Sirt3, we transfected HT22 cells with lentivirus that knocked down Sirt3 expression. As shown in Fig. 8A, Sirt3 expression was decreased by 58.58% (Sirt3 shRNA vs. negative control [NC], *p* < 0.001; Fig. 8A); PACAP treatment could not restore its expression (Sirt3 shRNA + PACAP vs. Sirt3 shRNA alone, *p* = 0.710). In Sirt3-knockdown cells exposed to 20 μM oligomeric Aβ42, Aβ and Tau deposition could not be reversed by PACAP treatment (Fig. 8B, C). Sirt3 knockdown caused Tau deposition independent of oligomeric Aβ42 exposure (28.16% Tau deposition in Sirt3 shRNA cells vs. NC cells, *p* = 0.038; Fig. 8C). In Sirt3-knockdown cells exposed to 20 μM oligomeric Aβ42, PACAP treatment did not improve the LC3-II:I ratio (Sirt3 shRNA + Aβ42 + PACAP vs. Sirt3 shRNA + Aβ42, *p* = 0.503; Fig. 8D). However, Sirt3-knockdown cells displayed a 13.70% increase in the LC3-II:I ratio, compared with NC cells (*p* = 0.036; Fig. 8D). Changes in autophagy were confirmed by LC3 turnover assays. The ratio of LC3-II:I was lower in CQ-treated Sirt3-knockdown cells than in CQ-treated NC cells (22.09% decrease vs. NC + CQ, *p* = 0.006; Fig. 8E). This result suggested that autophagy was diminished in the absence of Sirt3; it could not be restored by PACAP treatment. To clarify the pathway by which Sirt3 deficiency hinders autophagy, we measured proteins associated with multiple autophagy pathways. Regardless of oligomeric Aβ42 exposure, downregulation of Sirt3 led to hyperactivation of the PI3K-AKT and mTOR pathways and eliminated the therapeutic effects of PACAP (*p* < 0.05; Fig. 8F, G, H).

### Autophagy was impaired in patients with AD

**Fig. 9:**
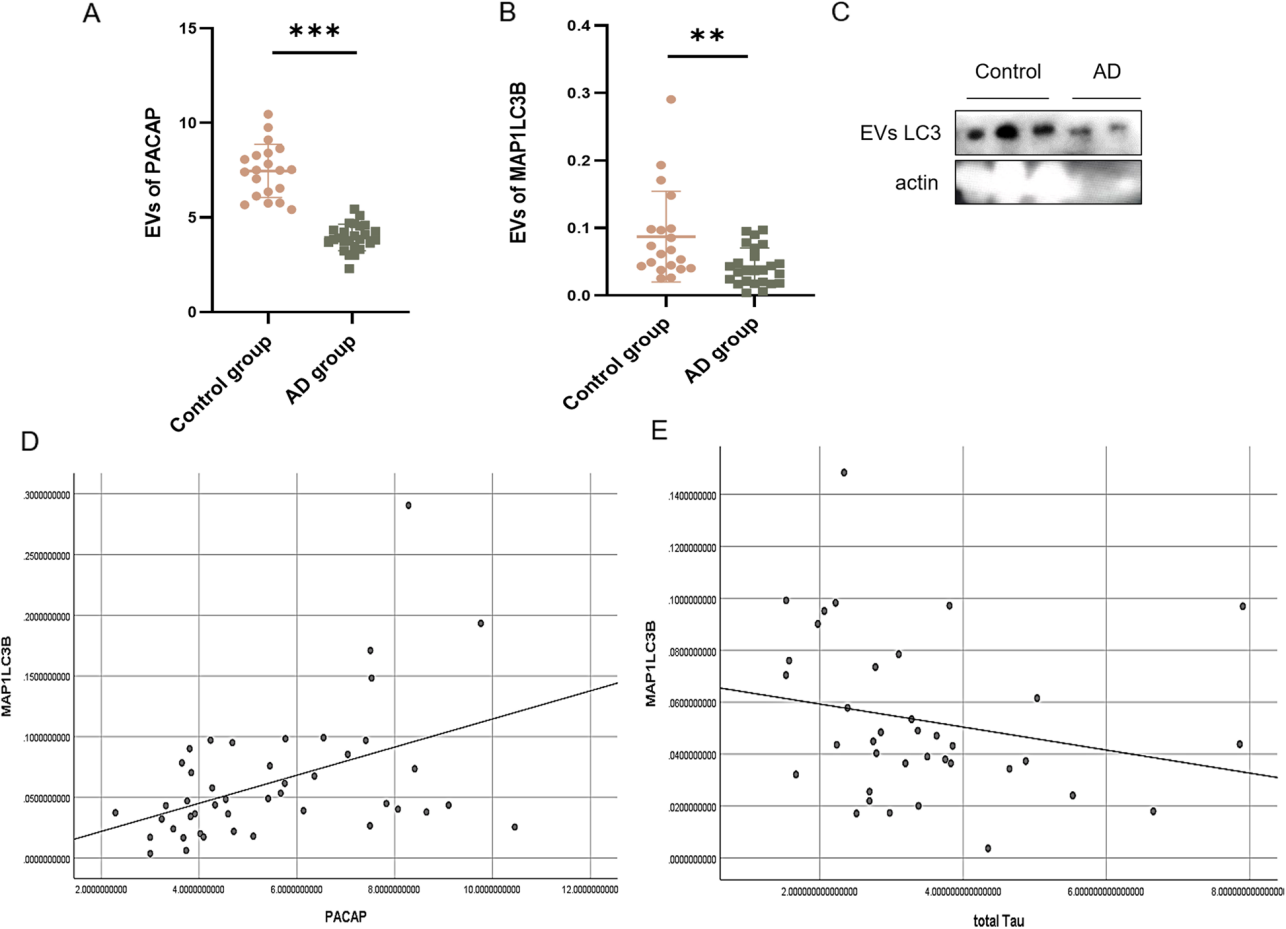
Autophagy and PACAP were decreased in patients with AD. Graphs show protein levels of (**A**) PACAP and (**B**) MAP1LC3B extracted from human serum exosomes (exosomal vesicles [EVs]), as determined by enzyme-linked immunosorbent assays. **C** Western blot of LC3 from human serum EVs (controls vs patients with AD). **D** and **E** Scatter plots of linear correlation analysis between MAP1LC3B and PACAP or total Tau. **p < 0.01, ***p < 0.001

As shown in Table 1, the CDR Global Scores were 0 in cognitively normal individuals and ≥ 0.5 in patients with AD (*p* < 0.001). Plasma pTau concentrations were increased in patients with AD (59.03% increase vs. cognitively normal individuals, *p* = 0.004), whereas plasma Aβ concentrations were decreased (19.90% decrease vs. cognitively normal individuals, *p* = 0.028). Serum exosomal concentrations of PACAP and MAP1LC3B in patients with AD were decreased by 47.18% and 50.57%, respectively, compared with concentrations in cognitively normal individuals. The linear regression results also revealed the differences of PACAP and MAP1LC3B in AD and control groups (Table 2). These findings were consistent with results in mice and cell-based assays (*p* < 0.01; Fig. 9A, B, C). Enzyme-linked immunosorbent assays of related proteins and linear correlation analysis indicated that MAP1LC3B and PACAP concentrations were positively correlated (*p* = 0.002; Fig. 9D); MAP1LC3B and total Tau concentrations were negatively correlated (*p* = 0.040; Fig. 9E). These results suggested that autophagy is impaired in patients with AD; this impairment is associated with decreased PACAP levels and enhanced deposition of Tau.

**Table 1:** The demographic characteristics for AD group and control group patients

	Total (*n* = 45)	Control group (*n* = 20)	AD group (*n* = 25)	*P* value
Age (years)	62.53 ± 9.68	58.25 ± 8.88	65.96 ± 9.04	0.006*
Sex, n (%) Female	26 (57.78%)	11 (55.00%)	15 (60.00%)	0.739
Education (years)	12.00 ± 3.84	12.25 ± 3.45	11.80 ± 4.18	0.847
PiB PET-CT, n (%) (positive)	25 (55.6%)	0 (0.00%)	25 (100%)	< 0.001*
CDR Global Score	0.71 ± 0.87	0.00 ± 0.00	1.28 ± 0.79	< 0.001*
Blood biomarkers				
PACAP	5.51 ± 2.06	7.46 ± 1.41	3.94 ± 0.70	< 0.001*
MAP1LC3B	0.063 ± 0.054	0.087 ± 0.067	0.043 ± 0.028	0.003*
Aβ42	10.26 ± 3.18	11.71 ± 3.69	9.38 ± 2.52	0.028*
pTau	4.91 ± 2.71	3.51 ± 2.09	5.82 ± 2.72	0.004*
Tau	3.44 ± 1.57	3.30 ± 1.57	3.62 ± 1.60	0.552

Values are mean ± standard deviation

^*^A significant statistic difference between AD group and control group patients at *p* < 0.05

All target proteins are from human plasma

**Table 2:** The linear regression of blood biomarkers

		Beta, 95%CI	*P* value
MAP1LC3B	Model 1	-0.004 [-0.007, -0.0004]	0.028
MAP1LC3B	Model 2	-0.004 [-0.007, -0.0005]	0.026
PACAP	Model 1	-0.359 [-0.430, -0.287]	< 0.001
PACAP	Model 2	-0.357 [-0.430, -0.284]	< 0.001

Model 1 controlled age

Model 2 controlled age and sex

## Discussion

In this study, compared with 3xTg untreated mice, 3xTg + PACAP mice displayed increased levels of ATG12-ATG5-ATG16 and VPS34, which are required for Beclin1 signaling [24]. ATG12-ATG5-ATG16 complex is essential to autophagy because it promotes the lipidation of LC3 [62, 63]. ATG4 is an autophagy activator that frees LC3-PE (ATG8-PE) from lipids and promotes autophagosome formation [64, 65].

RNA-seq analyses identified several pathways by which PACAP could promote autophagy in AD mice: PI3K-AKT, mTOR, and AMPK (Fig. 2). Inhibition of mTOR and PI3K-AKT pathways or enhancement of AMPK pathway can enhance autophagy [18–20, 66–69]. The in vivo and in vitro results in this study exhibited that PACAP treatment increased autophagic flux in these three pathways, leading to the clearance of Aβ, Tau, and pTau deposition, with subsequent attenuation of cognitive impairment. PACAP can activate AMPK function [70], and PACAP is proved to mediate the mTOR pathway via PAC-1 receptor activation in model of schizophrenia [71]. PI3K-AKT is also a key signal pathway affected by PACAP [72]. In our studies, we confirmed these signal pathways mediated by PACAP in AD mice.

PACAP is essential in the nervous system and has therapeutic effects in many diseases; however, further research is needed concerning the use of PACAP for formal treatment [41, 73, 74]. In AD, impaired autophagy results in Aβ and Tau deposition, followed by cognitive impairment [5, 7, 8, 75]. We found that PACAP treatment could not enhance autophagy or reduce Aβ/Tau deposition in Sirt3-knockdown HT22 cells. Our study demonstrated that PACAP may reduce Tau accumulation via Sirt3-dependent autophagy. Our team has proved the key function of Sirt3 on PACAP before [35, 36], knocking down Sirt3 abolished the protective function against Aβ toxicity of PACAP, and over-expressing of Sirt3 showed the contrary.

In this study, we found that the downregulation of Sirt3 alone in HT22 cells led to Tau deposition. Sirt3 deficiency could impair autophagic flux, causing accumulation of LC3-II (Fig. 8D, E). The activation function of Sirt3 on PI3K-AKT and mTOR pathways has been reported in lung cancer cells [76], which is similar in HT22 cells showed in our results. Regardless of oligomeric Aβ42 exposure, Sirt3 knockdown could activate the PI3K-AKT and mTOR pathways, disrupting autophagic balance (Fig. 8F, G, H). That reveals that PACAP-Sirt3 pathway is crucial in mediating PI3K-AKT and mTOR pathway, and PACAP may medicate autophagy mainly through Sirt3.

Our analysis of samples from patients with AD revealed that autophagy was altered in those patients, similar to findings in our animal model and cell-based assays. Although our analysis included a small number of patient samples, it provided insight concerning the potential for autophagy as an AD treatment target. We have described multiple models, including patient samples and preclinical biomarker data, along with sensitive assays for Aβ, tau, and exosomal protein contents in human serum. However, because of limitations regarding animal models, validation experiments are needed.

PACAP treatment attenuated cognitive disorder through autophagy in a Sirt3-dependent manner. Our animal model and cell-based assays revealed three pathways with possible involvement. However, it is unclear whether PACAP–Sirt3 signaling affects autophagy solely through these three pathways. Notably, because we did not conduct analyses of Sirt3 knockout mice, there is insufficient evidence to confirm that PACAP–Sirt3 directly impacts the three pathways, despite the RNA-seq findings. Thus, further studies should be performed in animal models. Cell-based assays showed that Sirt3 knockdown altered the expression patterns of the three pathways; nevertheless, other links between PACAP and the three pathways may exist. Additional cell-based assays are needed to determine whether PACAP–Sirt3 restores autophagy through these pathways; other cell lines should also be used to validate the findings.

Finally, some studies have shown that an increase in the p-AKT:AKT ratio is associated with beneficial effects, but an excessively high ratio is associated with autophagy impairment, neuronal synaptic loss, and cognitive decline [19, 20]. In this study, we found that PACAP decreased the p-AKT:AKT ratio in 3xTg + PACAP mice compared with 3xTg untreated mice; additionally, the p-AKT:AKT ratio was higher in 3xTg + PACAP mice than in WT mice (*p* = 0.019; Fig. 7B). We speculate that PACAP has a moderate ability to control the p-AKT:AKT ratio. Nevertheless, these results were observed in the animal model and could not be replicated in cell-based assays. In HT22 cells, there was no significant difference between the control and Aβ + PACAP groups (*p* = 0.410; Fig. 7G). The discrepancy between in vitro and in vivo results requires further investigation.

## Conclusions

This study provided insights concerning potential treatments for AD and showed novel AD-related signaling pathways that involve PACAP. In particular, autophagy may be restored by PACAP, reversing cognitive decline in AD; Sirt3 downregulation would reduce the therapeutic effect of PACAP.

### Supplementary Information

**Additional file 1:** **Fig. S1.** Flow chat about autophagy and building of animal model. **Fig. S2. **PACAP alleviated the anxiety of AD mice. **Fig. S3.** Other significant changes in mRNA profiles. **Supplementary Table S1. **Primer sequence. **Supplementary Table S2. **Antibodies used in western blot. **Supplementary Table S3. **The ApoE genotype of Control group and AD group.

**Additional file 2.** The detailed mRNA data.

**Additional file 3.** Full western blots.

## Data Availability

The datasets used or analyzed during the current study are available from the corresponding author on reasonable request.

## Abbreviations

AD: Alzheimer’s disease
AKT: Serine/threonine kinase
ATG: Autophagy-related
DEGs: Differentially expressed genes
AMPK: 5’Adenosine monophosphate-activated protein kinase
CCK-8: Cell counting kit-8
EVs: Extracellular vesicles or exosomes
CQ: Chloroquine
ELISA: Enzyme-linked immunosorbent assay
GO: Gene Ontology
HT22: Mouse hippocampal neuronal cell line
KEGG: Kyoto Encyclopedia of Genes and Genomes
MAP1LC3/LC3: Microtubule-associated protein 1 light chain 3
MWM: Morris water maze
mTOR: Mechanistic target of rapamycin kinase
P-AMPK: Phosphorylated AMPK
PBS: Phosphate-buffered saline
P-AKT: Phosphorylated AKT
PI3K: Phosphatidylinositol-3-kinase
P-mTOR: Phosphorylated mTOR
P-PI3K: Phosphorylated PI3K
RT-qPCR: Real-time quantitative polymerase chain reaction
RNA-seq: RNA-sequencing
SGD: Serum and glucose deprivation
shRNA: Short hairpin RNA
TBST: Tris-buffered saline plus 0.1% Tween
TEM: Transmission Electron Microscopy
3xTg mice: APP/PS1/tau triple-transgenic mice
WT: Wild type
