# Supplementary material for: PACAP–Sirtuin3 alleviates cognitive impairment through autophagy in Alzheimer’s disease
Source: Alzheimers Res Ther. 2023 Oct 27;15:184. doi: 10.1186/s13195-023-01334-2 (PMC10605376; doi:10.1186/s13195-023-01334-2)
Supplement: Supplementary file 1 — Additional file 1: Fig. S1. Flow chat about autophagy and building of animal model. Fig. S2. PACAP alleviated the anxiety of AD mice. Fig. S3. Other significant changes in mRNA profiles. Supplementary Table S1. Primer sequence. Supplementary Table S2. Antibodies used in western blot. Supplementary Table S3. The ApoE genotype of Control group and AD group. [file 13195_2023_1334_MOESM1_ESM.pdf]

# Supplementary materials

## Supplementary Figure S1

A

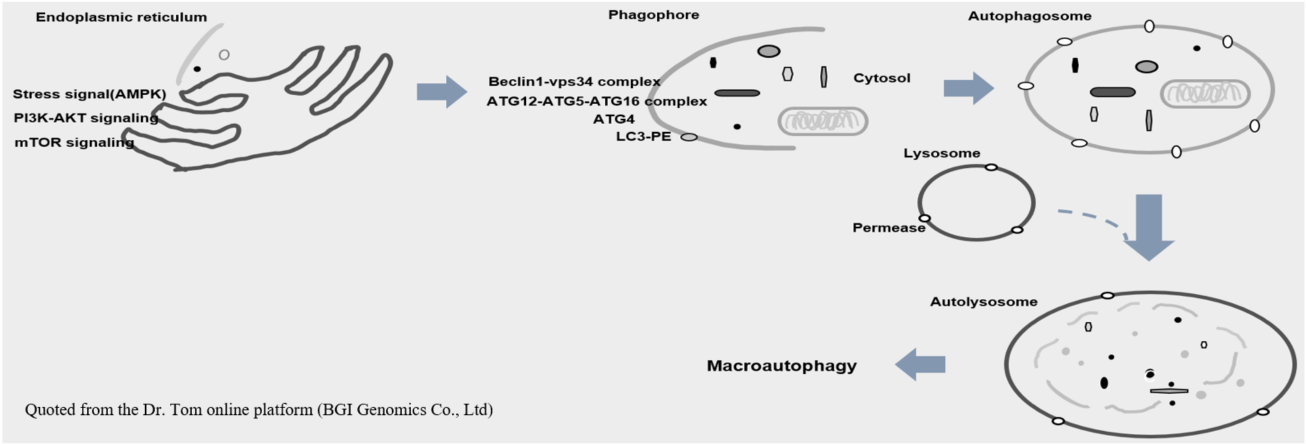


B

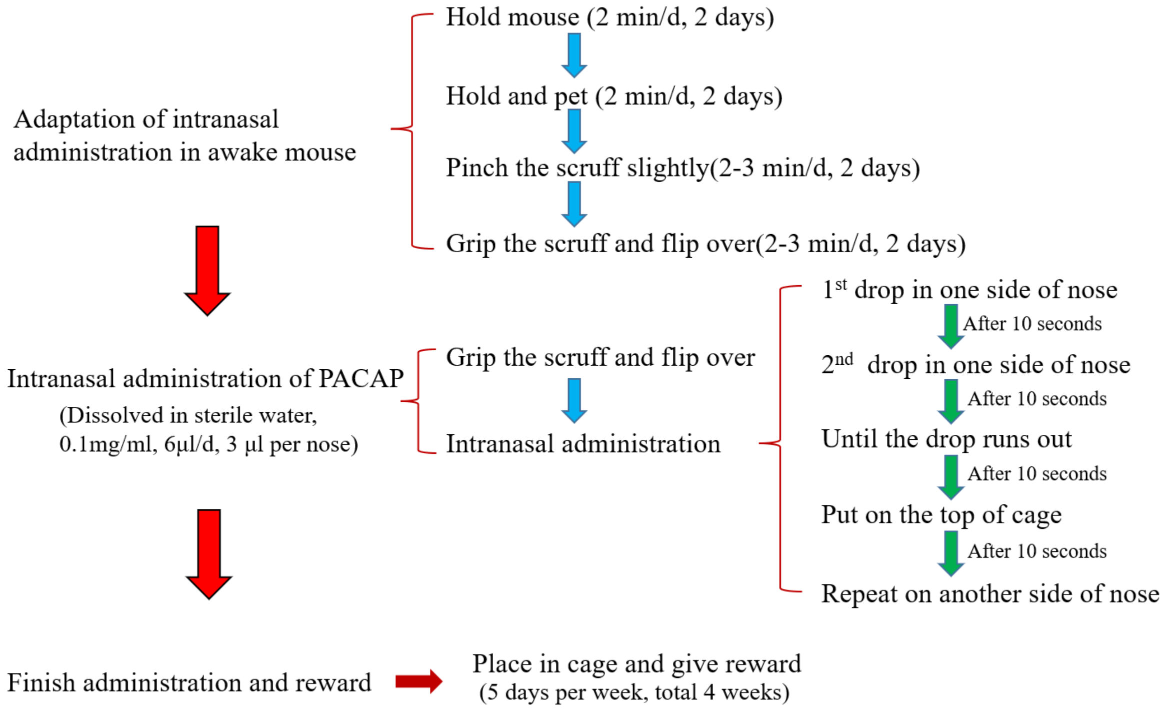


**Fig. S1** Flow chat about autophagy and building of animal model. (**A**)The procedure of autophagy from autophagosome to autolysosome as well as some key proteins. The information included help us conduct the further study to detect the exact association between PACAP and autophagy in AD. (**B**)The detailed steps of intranasal administration of mice.

**Supplementary Figure S2**

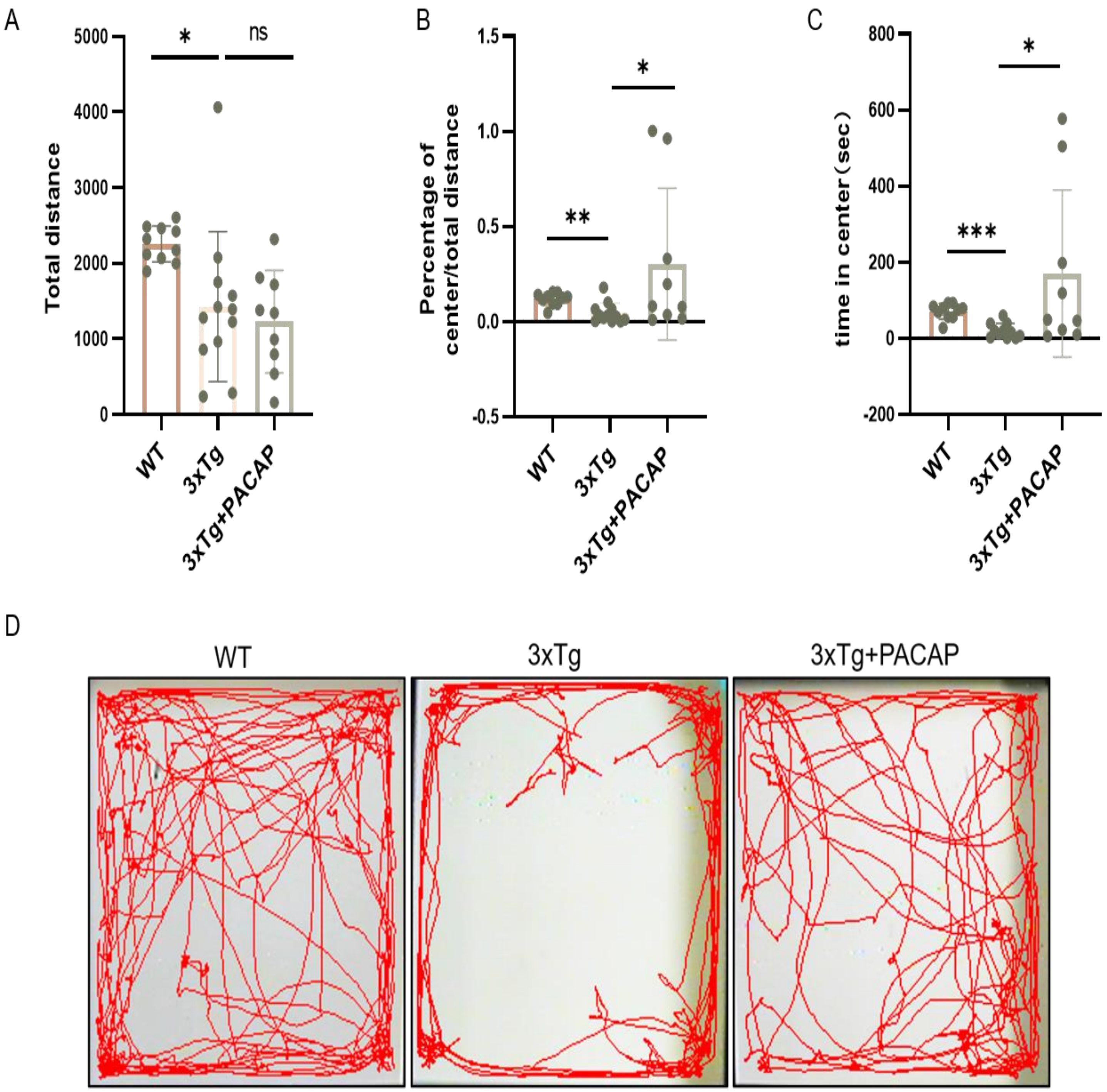


**Fig. S2** PACAP alleviated the anxiety of AD mice. (**A**)The total distance in open filed during 10 min among three groups. (**B**)Percentage of the central locus distance to the total peripheral distance of mice in open field, and the smaller the percentage, the more anxious it proved. (**C**) The amount of time mice spent in the middle of the field in 10 min, short time indicates anxiety. (**D**) Action trajectories of mice in the open filed. (*p<0.05, **p<0.01, ***p<0.001).

**Supplementary Figure S3**

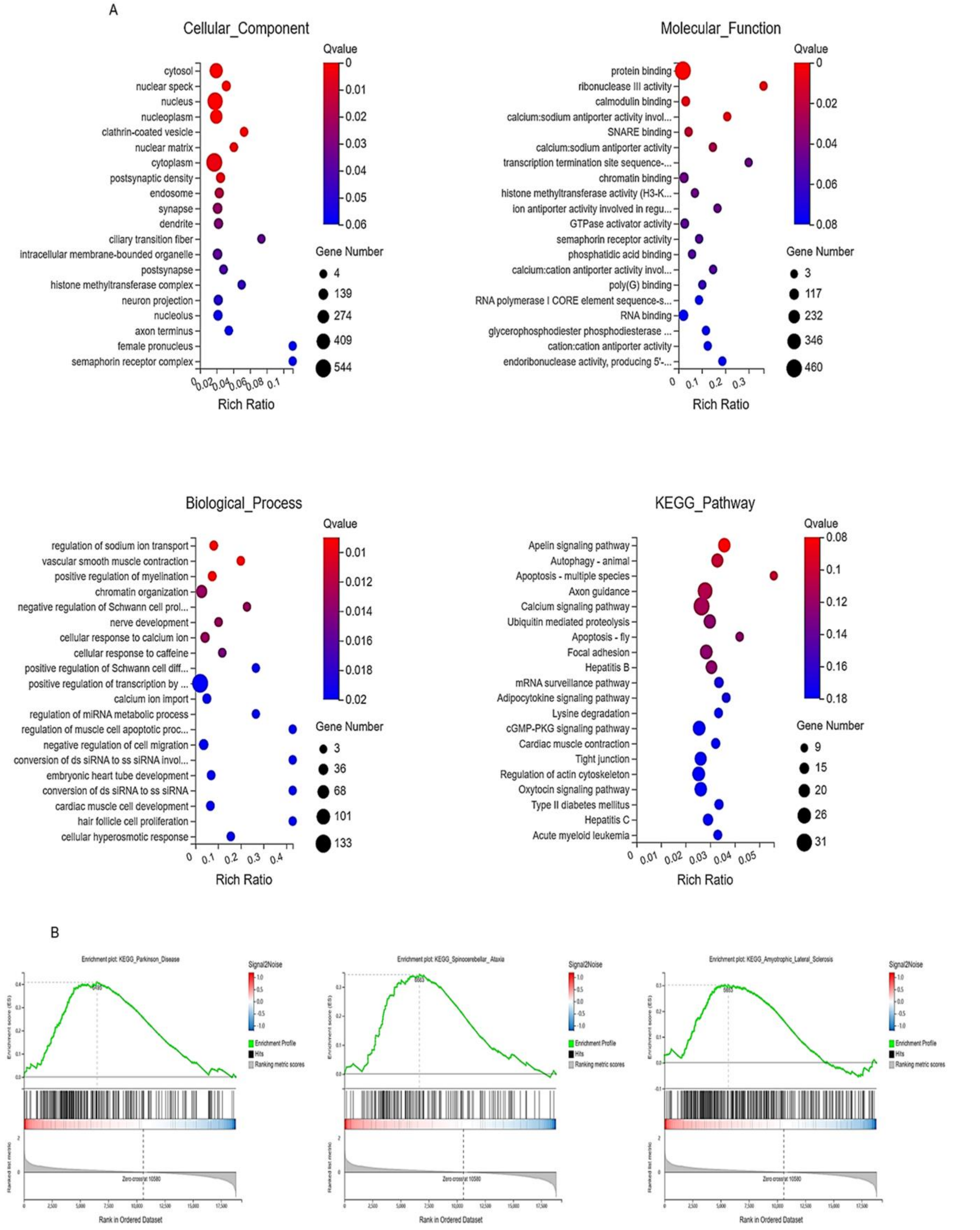


**Fig. S3** Other significant changes in mRNA profiles. (**A**) 20 kinds of GO and KEGG terms (**B**) Three KEGG pathways for neurodegenerative disease performed by Gene Set Enrichment Analysis (GSEA).

**Supplementary Table S1** Primer sequence

| Gene | Primer | Sequence |
|---|---|---|
| ATG5 | Forward | GCTTTTGCCAAGAGTCAGCTAT |
|  | Reverse | AACCAATTGGATAATGCCATTTCAG |
| Beclin1 | Forward | AGGAACTCACAGCTCCATTACT |
|  | Reverse | CTCTCCTGAGTTAGCCTCTTCC |
| GAPDH | Forward | GACCCCTTCATTGACCTCAAC |
|  | Reverse | TTCTCCATGGTGGTGAAGA |

**Supplementary Table S2** Antibodies used in western blot

| Target | Supplier | Catalog Number | Concentration |
|---|---|---|---|
| Aβ1-16 | Biolegend | 803001 | 1: 1000 |
| Tau-5 | BD Biosciences | 556319 | 1: 1000 |
| PACAP | Invitrogen | ZP006 | 1: 1000 |
| Map1LC3 | Cell Signaling Technology | 4108s | 1: 1000 |
| Atg5 | Cell Signaling Technology | 2630s | 1: 1000 |
| Beclin1 | Cell Signaling Technology | 3738s | 1: 1000 |
| Sirt3 | Cell Signaling Technology | 5490s | 1: 1000 |
| PI3K | Cell Signaling Technology | 4249s | 1: 1000 |
| P-PI3K | Cell Signaling Technology | 4228s | 1: 1000 |
| AKT | Cell Signaling Technology | 9272s | 1: 1000 |
| P-AKT | Cell Signaling Technology | 4060s | 1: 1000 |
| mTOR | Cell Signaling Technology | 2983s | 1: 1000 |
| p-mTOR | Cell Signaling Technology | 2971s | 1: 1000 |
| AMPK | Cell Signaling Technology | 2793s | 1: 1000 |
| p-AMPK | Cell Signaling Technology | 2535s | 1: 1000 |
| Beta Actin | Santa Cruz Biotechnology | sc-47778 | 1: 5000 |
| Goat Anti-Rabbit IgG | ComWin Biotech | CW0103S | 1: 5000 |
| Goat Anti-Mouse IgG | ComWin Biotech | CW0102S | 1: 5000 |

**Supplementary Table S3** The ApoE genotype of Control group and AD group

| **Control group** | **Genotype** | **AD group** | **Gene type** |
|---|---|---|---|
| Control group #1 | ε3/ε3 | AD group #1 | ε3/ε3 |
| Control group #2 | ε3/ε4 | AD group #2 | ε3/ε3 |
| Control group #3 | ε3/ε4 | AD group #3 | ε3/ε3 |
| Control group #4 | ε3/ε4 | AD group #4 | ε4/ε4 |
| Control group #5 | #N/A | AD group #5 | ε3/ε4 |
| Control group #6 | ε3/ε3 | AD group #6 | #N/A |
| Control group #7 | ε4/ε4 | AD group #7 | ε3/ε4 |
| Control group #8 | #N/A | AD group #8 | ε3/ε4 |
| Control group #9 | ε3/ε3 | AD group #9 | ε3/ε3 |
| Control group #10 | ε3/ε3 | AD group #10 | ε3/ε4 |
| Control group #11 | ε3/ε3 | AD group #11 | ε2/ε3 |
| Control group #12 | ε3/ε3 | AD group #12 | ε3/ε4 |
| Control group #13 | ε2/ε3 | AD group #13 | #N/A |
| Control group #14 | ε3/ε3 | AD group #14 | ε3/ε4 |
| Control group #15 | ε3/ε3 | AD group #15 | ε3/ε4 |
| Control group #16 | ε3/ε3 | AD group #16 | ε3/ε4 |
| Control group #17 | ε3/ε4 | AD group #17 | ε2/ε4 |
| Control group #18 | ε3/ε3 | AD group #18 | ε3/ε4 |
| Control group #19 | ε3/ε4 | AD group #19 | ε4/ε4 |
| Control group #20 | ε3/ε3 | AD group #20 | ε3/ε3 |
| | | AD group #21 | #N/A |
| | | AD group #22 | ε3/ε3 |
| | | AD group #23 | #N/A |
| | | AD group #24 | ε3/ε3 |
| | | AD group #25 | #N/A |
